# Joint diffusional kurtosis magnetic resonance imaging analysis of white matter and the thalamus to identify subcortical ischemic vascular disease

**DOI:** 10.1038/s41598-024-52910-x

**Published:** 2024-01-31

**Authors:** Min-Chien Tu, Sheng-Min Huang, Yen-Hsuan Hsu, Jir-Jei Yang, Chien-Yuan Lin, Li-Wei Kuo

**Affiliations:** 1 Department of Neurology, Taichung Tzu Chi Hospital, Buddhist Tzu Chi Medical Foundation, Taichung, Taiwan; 2 Department of Neurology, School of Medicine, Tzu Chi University, Hualien, Taiwan; 3 Institute of Biomedical Engineering and Nanomedicine, National Health Research Institutes, Miaoli, Taiwan; 4 Department of Psychology, National Chung Cheng University, Chiayi, Taiwan; 5 Center for Innovative Research on Aging Society (CIRAS), National Chung Cheng University, Chiayi, Taiwan; 6 Department of Radiology, Taichung Tzu Chi Hospital, Buddhist Tzu Chi Medical Foundation, Taichung, Taiwan; 7 GE Healthcare, Taipei, Taiwan; 8 Institute of Medical Device and Imaging, National Taiwan University College of Medicine, Taipei, Taiwan

**Keywords:** Neuroscience, Biomarkers, Neurology

## Abstract

Identifying subcortical ischemic vascular disease (SIVD) in older adults is important but challenging. Growing evidence suggests that diffusional kurtosis imaging (DKI) can detect SIVD-relevant microstructural pathology, and a systematic assessment of the discriminant power of DKI metrics in various brain tissue microstructures is urgently needed. Therefore, the current study aimed to explore the value of DKI and diffusion tensor imaging (DTI) metrics in detecting early-stage SIVD by combining multiple diffusion metrics, analysis strategies, and clinical-radiological constraints. This prospective study compared DKI with diffusivity and macroscopic imaging evaluations across the aging spectrum including SIVD, Alzheimer's disease (AD), and cognitively normal (NC) groups. Using a white matter atlas and segregated thalamus analysis with considerations of the pre-identified macroscopic pathology, the most effective diffusion metrics were selected and then examined using multiple clinical-radiological constraints in a two-group or three-group paradigm. A total of 122 participants (mean age, 74.6 ± 7.38 years, 72 women) including 42 with SIVD, 50 with AD, and 30 NC were evaluated. Fractional anisotropy, mean kurtosis, and radial kurtosis were critical metrics in detecting early-stage SIVD. The optimal selection of diffusion metrics showed 84.4–100% correct classification of the results in a three-group paradigm, with an area under the curve of .909–.987 in a two-group paradigm related to SIVD detection (all *P* < .001). We therefore concluded that greatly resilient to the effect of pre-identified macroscopic pathology, the combination of DKI/DTI metrics showed preferable performance in identifying early-stage SIVD among adults across the aging spectrum.

## Introduction

Subcortical ischemic vascular disease (SIVD) is a common subtype of vascular cognitive impairment (VCI) in which cerebral vascular pathologies are spatially and pathologically stratified^1^. Identifying SIVD in older adults is important but challenging^2^. While white matter hyperintensities (WMHs) and lacunes within subcortical regions are pathognomonic in SIVD^1^, these imaging features are also observed in patients with Alzheimer’s disease (AD)^3^ and cognitively normal (NC) older adults^4^. To improve the detectability, exploring the value of advanced neuroimaging techniques is needed from both therapeutic and prognostic perspectives. Current diagnostic guidelines for dementia mostly describe neuroimaging features for individual dementia subtypes separately. Therefore, an optimized tool to assist in decision-making is needed, as clinicians often consider a list of differential diagnoses simultaneously in real-world practice. Among neuroimaging techniques, structural MRI has been shown to potentially be able to identify the presence of VCI among patients with dementia, and especially among those with AD^5–7^. Compared to VCI, the early detection of SIVD is even more challenging, as the macroscopic plethora of WMHs and/or lacunes generally evolve later. Recently, diffusional kurtosis imaging (DKI) has been used to quantify microstructural changes, and it has been shown to improve the diagnostic accuracy of early-stage SIVD^8–10^. To date, most DKI studies have focused on comparing cerebral microstructural changes among SIVD subgroups^9^ or with controls^10^, but few have compared SIVD with other dementia subtypes^8^. Moreover, it remains unclear which DKI metrics have the best performance in detecting SIVD amongst the aging spectrum. Importantly, the statistical power for differential diagnostics that is pivotal for clinical drug trials should be optimized by characterizing the underlying neurobiological heterogeneity^11^. Given that the efficacy of DKI metrics can vary according to the targeted brain regions, macroscopic pathology, and clinical constraints, this study aimed to (i) investigate the diagnostic ability of DKI through two kinds of analytic strategies, (ii) validate the results either by considering multiple clinical constraints indicative of the very early stage of SIVD, or the effect driven by macroscopic pathology, and (iii) determine the optimal diffusion metrics to detect SIVD with a two-group or three-group paradigm.

## Results

### Demographics

Table 1 shows the basic information of the participants. There were group effects in demographics including age, education, and symptom duration (all *P* < 0.001), but not gender (*P* = 0.224). Other significant post-hoc differences were noted in WMHs (i.e., Scheltens scale and WMH volume; both *P* < 0.001; SIVD > AD > NC), the number of lacunes and Hachinski Ischemic Score (both *P* < 0.001; SIVD > NC and SIVD > AD), and global cognition (i.e., Mini-Mental State Examination (MMSE) and Cognitive Abilities Screening Instrument (CASI); both *P* < 0.001; SIVD < NC and AD < NC).

**Table 1:** Demographic of the participants.

	SIVD	AD	NC	*F/χ*^2§^	*P*
	(*N* = 42)	(*N* = 50)	(*N* = 30)		
**Age (year)**	73.5 ± 8.71	77.9 ± 5.05	70.5 ± 6.25	11.969 ^ac^	< .001
**Education (year)**	5.5 ± 4.47	4.9 ± 4.62	9.4 ± 4.14	10.101 ^bc^	< .001
**Symptom duration (year)**	1.5 ± 1.56	2.5 ± 2.22	0 ± 0.00	15.996 ^abc^	< .001
**Scheltens scale**	24.7 ± .7.52	12.0 ± 6.59	7.3 ± 6.68	63.791 ^abc^	< .001
**White matter hyperintensities (ml)**	38.8 ± 17.85	15.2 ± 14.64	6.3 ± 9.28	49.072 ^abc^	< .001
**Lacunes (number)**	20.4 ± 13.75	8.6 ± 8.94	7.7 ± 8.48	17.616 ^ab^	< .001
**Hachinski Ischemic Score**	10.0 ± 2.46	1.8 ± 1.45	1.9 ± 1.39	268.502 ^ab^	< .001
**Cognitive Abilities Screening Instrument**	63.3 ± 14.55	59.1 ± 18.25	89.9 ± 4.58	44.572 ^bc^	< .001
**Mini-Mental State Examination**	19.9 ± 5.03	19.1 ± 5.40	28.5 ± 1.07	43.875 ^bc^	< .001
**Gender (male/female)** **(***N***)**	19/23	16/34	15/15	2.991	.224
**Clinical Dementia Rating stage 0.5/1/2** **(***N***)**	25/12/5	22/24/4	–	3.634	.162

Data is represented as mean ± SD unless otherwise indicated.

SIVD = Subcortical ischemic vascular disease. AD = Alzheimer's disease. NC = normal cognition.

^§^The Analysis of Variance (ANOVA) and Chi-square test are used wherever appropriate. Significant between-group differences are remarked as ^a^SIVD vs. AD; ^b^SIVD vs. NC; ^c^AD vs. NC. Median of white matter hyperintensities (SIVD/AD/NC/overall) = (37.4/10.2/2.2/14.2) ml. Median of the lacune number (SIVD/AD/NC/overall) = (17/7/4/8).

### Diffusion metrics in the thalamus and white matter (WM)

Table 2 shows comparisons of diffusion metrics in the thalamus and WM after controlling for potential covariates including age, education, symptom duration, WMH volumes, and number of lacunes. Significant post-hoc results included: (i) in the thalamus, the SIVD group showed significantly lower fractional anisotropy (FA) and axial kurtosis (K_axial_) than the AD group (*P* = 0.011–0.041), and significantly lower mean kurtosis (MK) and radial kurtosis (K_radial_) than the NC group (*P* = 0.043–0.044); the AD group showed significantly higher mean diffusivity (MD), axial diffusivity (D_axial_), and radial diffusivity (D_radial_) than the NC group (*P* = 0.003–0.019), and (ii) in WM, the SIVD group showed significantly lower bilateral MK and K_radial_ (*P* = 0.004–0.019) than the NC group.

**Table 2:** Diffusional kurtosis and diffusion tensor metrics from the average thalamus and the white matter measures by groups.

Metrics	Average white matter atlas				Average thalamus			
	SIVD	AD	NC	ANCOVA	SIVD	AD	NC	ANCOVA
	(*N* = 42)	(*N* = 50)	(*N* = 30)	*P*	(*N* = 42)	(*N* = 50)	(*N* = 30)	*P*
**MK**	.88 ± .071	.99 ± .606	1.05 ± .083	***.004b***	.81 ± .063	.89 ± .074	.92 ± .048	***.031b***
**K**_**axial**_	.44 ± .030	.45 ± .025	.46 ± .021	.112	.45 ± .044	.44 ± .043	.45 ± .023	***.014a***
**K**_**radial**_	1.16 ± .123	1.33 ± .098	1.41 ± .127	***.019b***	.97 ± .087	1.0 ± .088	1.1 ± .068	***.037b***
**KFA**	.35 ± .063	.38 ± .046	.39 ± .034	.866	.28 ± .087	.32 ± .069	.32 ± .038	.796
**MD**	1.20 ± .110	1.10 ± .068	1.03 ± .069	.102	1.32 ± .256	1.18 ± .207	.98 ± .093	***.010c***
**D**_**axial**_	1.60 ± .093	1.52 ± .066	1.46 ± .061	.117	1.65 ± .281	1.53 ± .233	1.29 ± .100	***.003c***
**D**_**radial**_	1.00 ± .123	.90 ± .072	.82 ± .075	.123	1.15 ± .245	1.01 ± .198	.823 ± .092	***.019c***
**FA**	.31 ± .039	.35 ± .024	.37 ± .030	.182	.27 ± .035	.30 ± .035	.30 ± .024	***.047a***

Data is represented as mean ± SD unless otherwise indicated. Mean/axial/radial diffusivity is presented in units of 10^−3^mm^2^s^−1^. *P* value from the analysis of covariance (ANCOVA) with covariates including age, education, symptom duration, the volume of white matter hyperintensities, and the number of lacunes are reported. The Tukey post-hoc comparisons are remarked as **a**: SIVD vs. AD; **b**: SIVD vs. NC; **c**: AD vs. NC.

FA = Fractional Anisotropy. KFA = Kurtosis Fractional Anisotropy. MD = Mean Diffusivity. D_radial_ = Radial Diffusivity. D_axial_ = Axial Diffusivity. MK = Mean Kurtosis. K_radial_ = Radial Kurtosis. K_axial_ = Axial Kurtosis. SIVD = Subcortical ischemic vascular disease. AD = Alzheimer's disease. NC = normal cognition.

Significant values are in bolditalics.

### Diffusion metrics quantified in the white matter atlas (WMA) and segregated thalamus analysis (THA)

Table 3 shows the correct classification through discriminant analysis among the participants across the aging spectrum by diffusion metrics and macroscopic imaging evaluation in WMA and THA. At first glance, THA generally had a lower correct classification rate than WMA, but the trend among the performance of various diffusional kurtosis metrics was similar to that in WMA. Compared to correct classification by Scheltens scale (72.1%) and segregated WMH volume (63.9%), the use of mean kurtosis metrics in overall WMA achieved the highest correct classification rate (77.9%). Generally, FA showed higher correct classification rates than kurtosis fractional anisotropy (KFA) in all constraints except CDR. MK showed incrementally higher classification rates over MD in all cases (overall and three constraints) for both WMA and THA analyses. For both diffusivity and kurtosis, the mean and radial metrics generally showed higher correct classification rates than the axial metrics. Since KFA, D_axial_, and K_axial_ showed relatively low correct classification rates, they were excluded from comparisons of discrimination analyses using multiple metrics. Given a generally better performance in the measure of saving area of WMHs than the current measure aimed at region *free* from WMH, the statistical differences are not benefited by the process of WMH regional exclusion (Supplementary Table S1).

**Table 3:** Discriminant analysis by diffusional kurtosis metrics, diffusion tensor metrics, and macroscopic imaging evaluation.

Metrics	White matter atlas*				Segregated thalamus analysis^§^			
	Overall	CDR ≤ .0.5	WMH ≤ 15 ml	Lacune ≤ 8	Overall	CDR ≤ .0.5	WMH ≤ 15 ml	Lacune ≤ 8
**Scheltens scale**^†^	72.1	72.7	67.2	72.6	**–**	**–**	**–**	**–**
Segregated WMH volume*	63.9	75.3	85.2	72.6	**–**	**–**	**–**	**–**
**MK**	77.9	81.8	81.0	77.4	68.9	76.6	80.3	79.0
**K**_**axial**_	68.9	76.6	73.8	72.6	56.6	64.9	59.0	56.5
**K**_**radial**_	74.6	80.5	78.7	83.9	66.4	64.9	70.5	69.4
**KFA**	66.4	75.3	78.7	75.8	58.2	63.6	70.5	66.1
**MD**	76.2	80.5	80.3	77.4	63.1	68.8	75.4	71.0
**D**_**axial**_	76.2	76.6	78.7	74.2	55.7	67.5	73.8	74.2
**D**_**radial**_	71.3	77.9	88.5	79.0	65.6	76.6	73.8	74.2
**FA**	72.1	75.3	88.5	79.0	66.4	72.7	77.0	69.4

Data are reported by the value of correct classification (%). CDR = Clinical Dementia Rating. WMH = white matter hyperintensities. FA = fractional anisotropy. MD = mean diffusivity. MK = mean kurtosis. KFA = kurtosis fractional anisotropy. D_axial_ = axial diffusivity. D_radial_ = radial diffusivity. K_axial_ = axial kurtosis. K_radial_ = radial kurtosis. SIVD = Subcortical ischemic vascular disease. AD = Alzheimer's disease. NC = normal cognition. Macroscopic imaging evaluation included the Scheltens scale and segregated WMH volume.

^†^A total of 12 subregions averaged from bi-hemispheric ratings are used.

*A total of 16 tracts including the corpus callosum (genu and body parts), and the anterior limb of internal capsule, anterior corona radiata, superior corona radiata, external capsule, cingulum (cingulate gyrus part), and superior longitudinal fasciculus of bi-hemispheres are used.

^§^A total of 14 sub-regions of the thalamus including the pulvinar, anterior, mediodorsal, ventral–lateral–dorsal, central, ventral-anterior, and ventral–lateral–ventral nuclei are used. Cases number (SIVD/AD/NC): Overall = 42/50/30; CDR ≤ 0.5 = 25/22/30; WMH Volume ≤ 15 ml = 3/32/26; Lacunes ≤ 8 = 8/34/20.

### Optimized selection of multiple diffusion metrics

**Figure 1:**
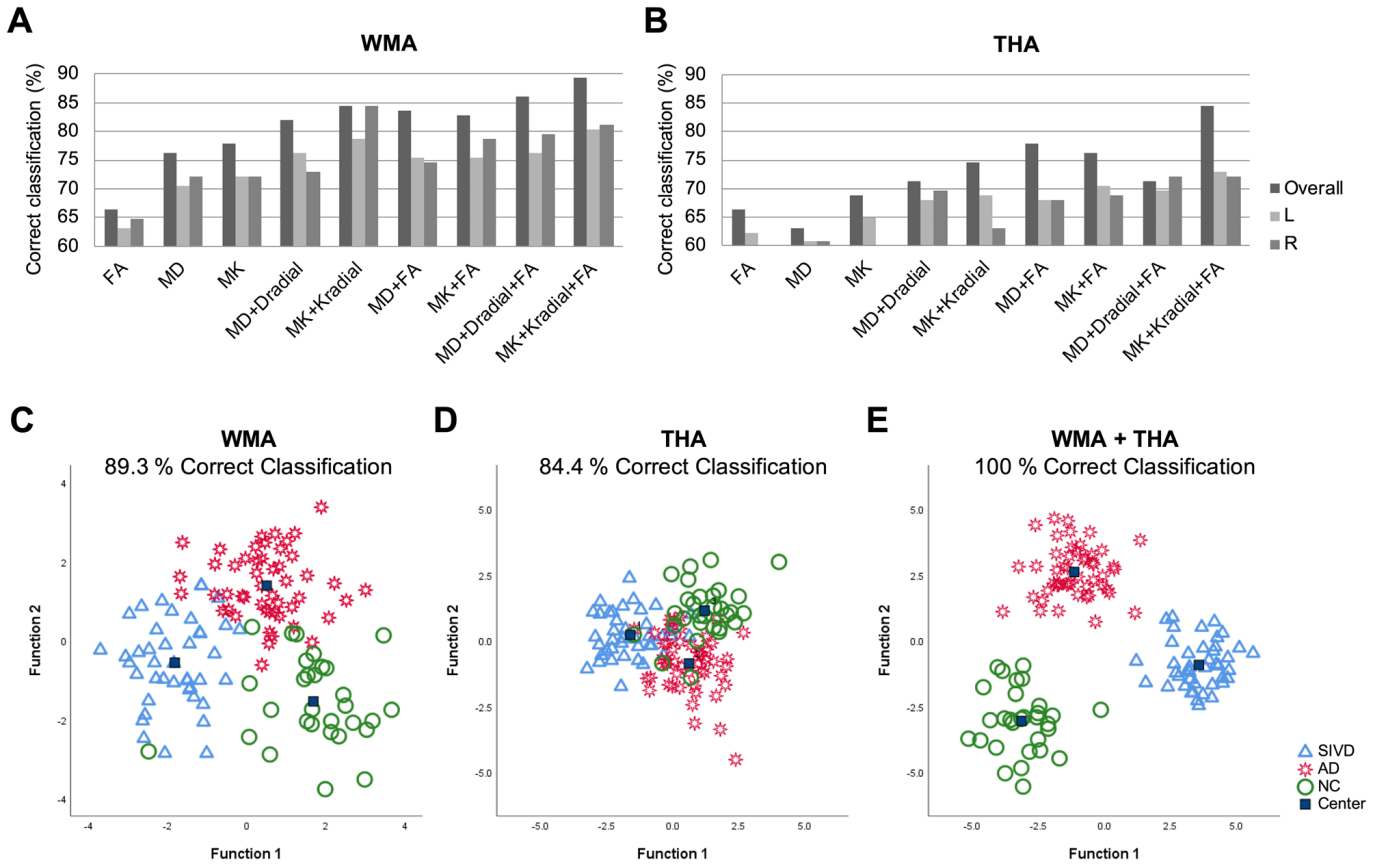
Discriminant analysis determined by kurtosis and diffusion metric selection. (**A**, **B**) Comparisons of variable metrics (e.g., MK, MD, and FA) by two measures (i.e., WMA and THA) shows that integration of triple metrics (MK + K_radial_ + FA) achieves the best discriminant analysis of aging spectrum including SIVD, AD, and NC (89.3% and 84.4% correct classification in the WMA and THA, respectively. In the WMA, the correct classification rate by single/double/triple metrics is 66.4–77.9/82.0–84.4/86.1–89.3%, with the averaged correct classification rate of 73.5/83.2/87.7%, respectively. In the THA, the correct classification rate by single/double/triple metrics is 63.1–68.9/71.3–77.9/71.3–84.4%, with the averaged correct classification rate of 66.1/75.0/77.9%, respectively. The correct classification rates of utilizing single/double/triple metrics were derived by entering the selected diffusion metrics (e.g. MK, MD, FA, …etc.) from white matter atlas or thalamic atlas in to discriminant analysis. Data from different hemispheres were regarded as different inputs. Taking a triple-metric (MK + MD + FA) as an example, such triple-metric in bilateral WMA (total 16 regions) will give 48 values in each individual, and these 48 values are entered into discriminant analysis. The optimized kurtosis metrics (i.e., MK + Kradial + FA) is further examined by deciphering primary regions of interest by hemispheres, showing 80.7 and 72.3% average correct classification in the WMA and THA, respectively. (**C**–**E**) Discriminant analysis results from the optimized kurtosis metrics are plotted, showing that 89.3% correct classification in the WMA, 84.4% correct classification in the THA, and 100.0% correct classification in WMA + THA. R/L = Right/Left hemisphere. FA = fractional anisotropy; MD = mean diffusivity; MK = mean kurtosis; D_radial_ = radial diffusivity; K_radial_ = radial kurtosis. WMA = White matter atlas. THA = Segregated thalamus analysis. SIVD = Subcortical ischemic vascular disease. AD = Alzheimer's disease. NC = normal cognition.

Figure 1 compares the performance of single diffusion metric and multiple diffusion metrics in discriminating the three groups. The correction classification rates of utilizing single/double/triple metrics from both hemispheres were 73.5/83.2/87.7% in WMA (Fig. 3A), and 66.1/75.0/77.9% in THA (Fig. 3B). In analysis of two hemispheres separately, the use of triple metrics showed correct classification rates of 80.7 and 72.3% in WMA and THA, respectively (Fig. 3A, B). Of note, the triple-metric usage of MK, K_radial_, and FA achieved the best discriminant power across the aging spectrum (89.3/84.4% correct classification in WMA/THA) (Fig. 3C, D). Importantly, the use of both analysis strategies (i.e., WMA + THA) yielded a 100.0% correct classification rate (Fig. 3E).

### ROC values from the optimal selection of triple diffusion metrics

**Figure 2:**
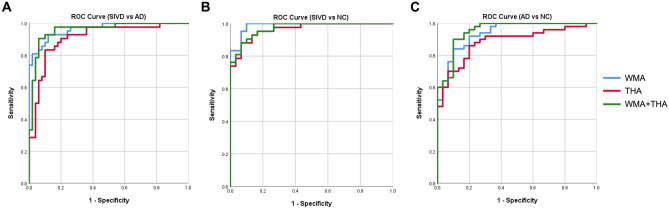
Optimized diffusional kurtosis metrics performance by receiver operating characteristic (ROC) curves. ROC curves were used to differentiating targeted diseased group. Ranked by AUC values, performance of optimized kurtosis metrics was displayed in (**A**) differentiating SIVD from AD by WMA [AUC = .964 (95% CI 0.932–0.997)], WMA + THA [AUC = .958 (95% CI 0.918–0.998)], and THA [AUC = .909 (95% CI 0.847–0.971)], (**B**) differentiating SIVD from NC by WMA [AUC = .987 (95% CI 0.969–1.000)], WMA + THA [AUC = .971 (95% CI 0.942–1.000)], and THA [AUC = .965 (95% CI 0.930–1.000)], and (**C**) differentiating AD from NC by WMA + THA [AUC = .953 (95% CI 0.908–0.997)], WMA [AUC = .937 (95% CI 0.886–0.988)], and THA [AUC = .885 (95% CI 0.881–0.958)]. WMA = White matter atlas. THA = Segregated thalamus analysis. SIVD = Subcortical ischemic vascular disease. AD = Alzheimer's disease. NC = normal cognition. WMA = White Matter Atlas. T = Segregated thalamus. ROC = Receiver operating characteristic curves. AUC = Area under curve. CI = Confidence interval.

Figure 2 shows the ROC curves for the sensitivity and specificity of optimal diffusion metrics to discriminate SIVD, AD and NC. To differentiate SIVD from AD, the highest AUC was for WMA (AUC = 0.964), followed by WMA + THA (AUC = 0.958) and THA (AUC = 0.909) (Fig. 4). To differentiate SIVD from NC (Fig. 4B), a similar trend was found (WMA > WMA + THA > THA; AUC = 0.987–0.965). Of note, all trials of the optimal DKI metrics aiming to detect SIVD yielded AUC values > 0.9. To differentiate AD from NC (Fig. 4C), WMA + THA achieved the best AUC of 0.953, followed by WMA (AUC = 0.937) and THA (AUC = 0.885) (all *P* < 0.001). Regarding individual regions, the tracts and nuclei showing the best statistical power were identified as (i) the left superior corona radiata and left ventral–lateral–ventral nuclei for SIVD vs. AD, (ii) the right anterior corona radiate and left ventral–lateral–ventral nuclei for for SIVD vs. NC, and (iii) the right cingulum (cingulate gyrus part) and left central nuclei for AD vs. NC. Note that all belong to MK metrics (Supplementary Table S2).

### Correlates for global cognition

Table 4 shows the estimated effects of diffusion metrics on global cognition measured using the CASI. In the WMA, the right cingulum, right genu of the corpus callosum, and bilateral superior corona radiata were the best predictors (*P* =  < 0.001–0.035). In THA, the nucleus including the left central, right ventral lateral-dorsal, right anterior, right mediodorsal, left pulvinar, and left ventral-anterior portions were significant (*P* =  < 0.001–0.023).

**Table 4:** Estimated effect (β coefficients) of diffusional kurtosis and diffusion tensor metrics on global cognition (*N* = 122).

Metrics	White matter atlas				Segregated thalamus analysis			
	Regions	β	*P*	95% CI	Regions	β	*P*	95% CI
**MK**	CIN_R	.199	.024	(6.880,94.025)	C_L	.287	< .001	(36.722, 110.999)
**K**_**axial**_	SCR_L	.222	.002	(24.535, 107.512)	VLD_R	.212	.002	(21.522, 96.746)
**K**_**radial**_	SCR_R	−.265	.001	(−39.707, −9.655)	C_L	.311	< .001	(37.771, 100.090)
	CIN_R	.232	.004	(9.148, 47.426)				
**KFA**	SCR_R	−.195	.006	(−129.004, −21.908)	A_R	−.159	.023	(−77.130, −5.943)
**MD**	GCC_R	−.232	.002	(−26.495, −6.319)	Medio_R	−.296	< .001	(−21.756, −7.146)
**D**_**axial**_	GCC_R	−.241	.001	(−29.636, −7.515)	P_L	−.375	< .001	(−46.005, −17.181)
**D**_**radial**_	CIN_R	−.190	.034	(−46.284, −1.853)	Medio_R	−.287	< .001	(−22.153, −6.917)
	GCC_R	−.161	.035	(−20.748, −0.755)				
**FA**	SCR_R	−.279	< .001	(−181.874, −59.624)	VA_L	−.210	.004	(−159.212, −31.029)
	CIN_R	.252	.002	(32.160,142.450)				

Significant regions are reported by stepwise linear regression analysis, with independent variables including targeted imaging metrics showing significant correlation with the total scores of the Cognitive Abilities Screening Instrument. Metrics with a Variance inflation factor (VIF) ≥ 5 are removed. Estimated effect of all metrics is reported after controlling for age, education, symptom duration, the volume of white matter hyperintensities, and the number of lacunes.

CIN = Cingulum. SCR = superior corona radiata. GCC = genu of the corpus callosum. A = anterior nuclei. C = central nuclei. P = pulvinar. VLD = ventral latero-dorsal. Medio = medio-dorsal. VA = ventral-anterior. R/L = Right/Left hemisphere. FA = fractional anisotropy. MD = mean diffusivity. MK = mean kurtosis. KFA = kurtosis fractional anisotropy. D_axial_ = axial diffusivity. D_radial_ = radial diffusivity. K_axial_ = axial kurtosis. K_radial_ = radial kurtosis. Mean/axial/radial diffusivity variables are entered in units of 10^−3^mm^2^s^−1^.

### Correlates for the Hachinski Ischemic Scale (HIS)

We also explored the associations between all diffusion metrics and the HIS. In the WMA, the bilateral anterior limbs of the internal capsule were the best predictors (*P* =  < 0.001–0.005). In THA, the nucleus including the bilateral ventral latero-ventral, right ventral latero-dorsal, and right ventral-anterior were the significant (*P* = 0.001–0.049) (Supplementary Table S3).

## Discussion

The current study shows the complementary value of using both diffusivity and kurtosis metrics. FA and DKI metrics in average THA could differentiate SIVD from AD and NC, and so could DKI metrics in average WMA. The selection of metrics was evaluated based on two analysis strategies (WMA and THA) and clinical-radiological constraints indicative of the very early stage of SIVD (e.g., WMH volume and number of lacunes). In the three-group discriminant analysis, we showed the power of DKI/DTI metrics in identifying SIVD. The optimal combination of metrics, i.e., MK, K_radial_, and FA, yielded the highest correct classification rate. This combination was then validated using two-group comparisons via ROC analysis, which showed promising results overall. In addition, several WMA and THA hubs with significant effects in predicting global cognition anatomically overlapped with Papez and frontal-subcortical circuits, supporting the robustness of the diffusion metrics in exploring cognitive and neuronal substrates.

Our findings demonstrated that both DKI and DTI metrics provided better classification accuracy than Scheltens scale and segmented WMH volume. In average ROI analysis, MK performed incrementally better than MD across various clinical constraints. This corroborates the sensitivity inherited by the non-Gaussian premise assumption in DKI. It was also noticeable that the radial components of both kurtosis and diffusivity generally outperformed their axial components in terms of classification accuracy. Previous studies have shown that MK and K_radial_ exhibited a greater extent and degree of SIVD–AD differences than K_axial_ within the WM^12^ and thalamus^8^. Another report has shown that radial metrics could possess higher sensitivity in detecting AD, mild cognitive impairment, and cognitively normal individuals^13^. As consistent with previous reports, the radial metrics could provide higher sensitivity in detecting the microstructural alterations especially in SIVD and AD. Hypothetically, liquefaction within the halo of lacunar/micro-infarcts and myelin pallor within WMHs^14^ could contribute to the geometrical variability of cerebral microstructures. We speculate that the attenuated discriminant power of K_axial_ may be due to diverse diffusing axis determination in the presence of neuronal and/or interstitial dispersion from vascular insults. This does not negate the importance of the axial components of kurtosis and diffusivity, and both K_axial_ and D_axial_ in our results could still differentiate SIVD or AD from NC. The importance of axial components was also suggested in a previously published AD cohort^15^. It is also worth mentioning that the classification rates of MK and MD improved remarkably with WMHs and lacunes as constraints in the thalamus rather than in WM. This effect of WMHs and lacunes could be associated with differences in regional vulnerability to vascular burden between WM and the thalamus, or the better inherited power of WMA than THA due to higher structural coherence within WM than the thalamus. Of note, independent evidence supports a viewpoint that the degree of DKI changes in response to ischemia differs according to the gray-white matter proportion of the affected regions^16^.

After controlling for demographics and macroscopic pathology including WMHs and lacunes, FA and DKI metrics enhanced SIVD-AD and SIVD-NC contrasts in the average THA, and DKI metrics solely enhanced SIVD-NC contrast in the average WMA. Aside from the reported role of FA in detecting vascular dementia^6^, our results further indicate that DKI is potentially resilient to the overall effect from WMHs and lacunes. It is also worth mentioning that DTI metrics enhanced AD-NC contrast in the average THA, demonstrating the complementary value of DKI and DTI metrics. KFA and FA had similar tendencies in most comparisons, but KFA showed a lower correct classification rate than FA. As the DKI metrics were derived from a WMA driven by FA, the possibility of pseudo-normalization of KFA values when the quantification process involves crossing fibers as documented issues in FA calculations should be considered^17^. The numeric accuracy of KFA may also have been overwhelmed since WM and microstructure coherence was already compromised by vascular burden, and hence it may be a less promising metric in terms of test–retest reliability^10^ and statistical power^9^.

In this study, we used a WMA *free* from WMHs while considering WMHs and lacunes as statistical covariates, which is a two-tier concept. First, measurements focusing on normal-appearing WM could provide information presumably corresponding to pathological alterations at the very early stage of SIVD. Second, the additional statistical considerations reduced the effect driven by vascular pathology, which had already been identified macroscopically and may have had an indirect effect due to Wallerian degeneration. Of note, our results bridge the published findings^8,18^ by highlighting that a joint analysis strategy is a favorable solution for constructing a dementia classifier across the aging spectrum. We consider that the joint analysis strategy integrating both gray and WM is an informative ensemble which can be used to stratify the subtype of dementia across the aging spectrum. This also mirrors the regional/tissue vulnerability inherently associated with individual dementia subtypes. As we only investigated two dementia subtypes and cognitively normal older adults, identifying dementia subtypes other than SIVD or AD falls beyond the scope of discussion.

Our results connect neuronal substrates associated with global cognition to some specific WM hubs, including the genu of the corpus callosum and cingulum of the right hemisphere, and the bilateral superior corona radiata. In addition, THA delineated a pervasive pattern scattered across multiple thalamic nuclei. The overall hubs identified in this study considerably overlapped with landmarks belonging to the Papez circuit or frontal-subcortical circuit^19^. As the interplay between these two circuits plays a critical role in harmonizing global cognition^19^, using diffusional kurtosis metrics could provide additional value in exploring neuronal substrates critical for cognition. In addition, the current metrics exhibit pervasive correlations with the HIS, and the significance remains in considering demographics and indices of cerebral small vessel disease. This corroborates that the diffusional kurtosis metrics can be regarded as potential biomarkers that mirror the composite vascular risk in the aging spectrum. The strength of this study is the integration of WMA and THA analysis strategies with several clinical-radiological constraints targeting the very early stage of SIVD. Diffusion metrics were derived and compared with macroscopic pathology including WMHs and lacunes, and the results showed that combining DKI and DTI metrics could provide robust differential power and resilience to predefined vascular lesions. Moreover, the optimal combination of diffusion metrics showed robust performance in two- or three-group comparisons, suggesting its potential for clinical applications. There are also several limitations to the study. Not all dementia subtypes were included, and the prevalence of dementia subtypes varies across medical settings. Including more dementia subtypes in future studies may provide an opportunity to investigate the robustness and improve the optimal selection of diffusion metrics. In addition, the insignificant differences of diffusional kurtosis metrics between AD and NC within WM could be related to the excluding the effect of WMHs by both imaging processing and statistics. Since the effect of WMHs has been minimized, we believe that the diffusion metrics used in this study provide a reliable reference for future application in differential diagnostics.

In conclusion, the current study investigates the sensitivity and resilience of diffusion metrics for macroscopic vascular lesions that are pathognomonic to SIVD. The optimal combination of diffusion metrics included MK, K_radial_, and FA, which could effectively differentiate SIVD from other groups across the aging spectrum in both WMA and THA strategies (AUC > 0.9). The joint analysis strategy showed the best performance. The additional and complementary values of the diffusion metrics were comprehensively explored as hubs for global cognition identified and anatomically overlapped with Papez and frontal-subcortical circuits.

## Methods

### Study design and participants

**Figure 3:**
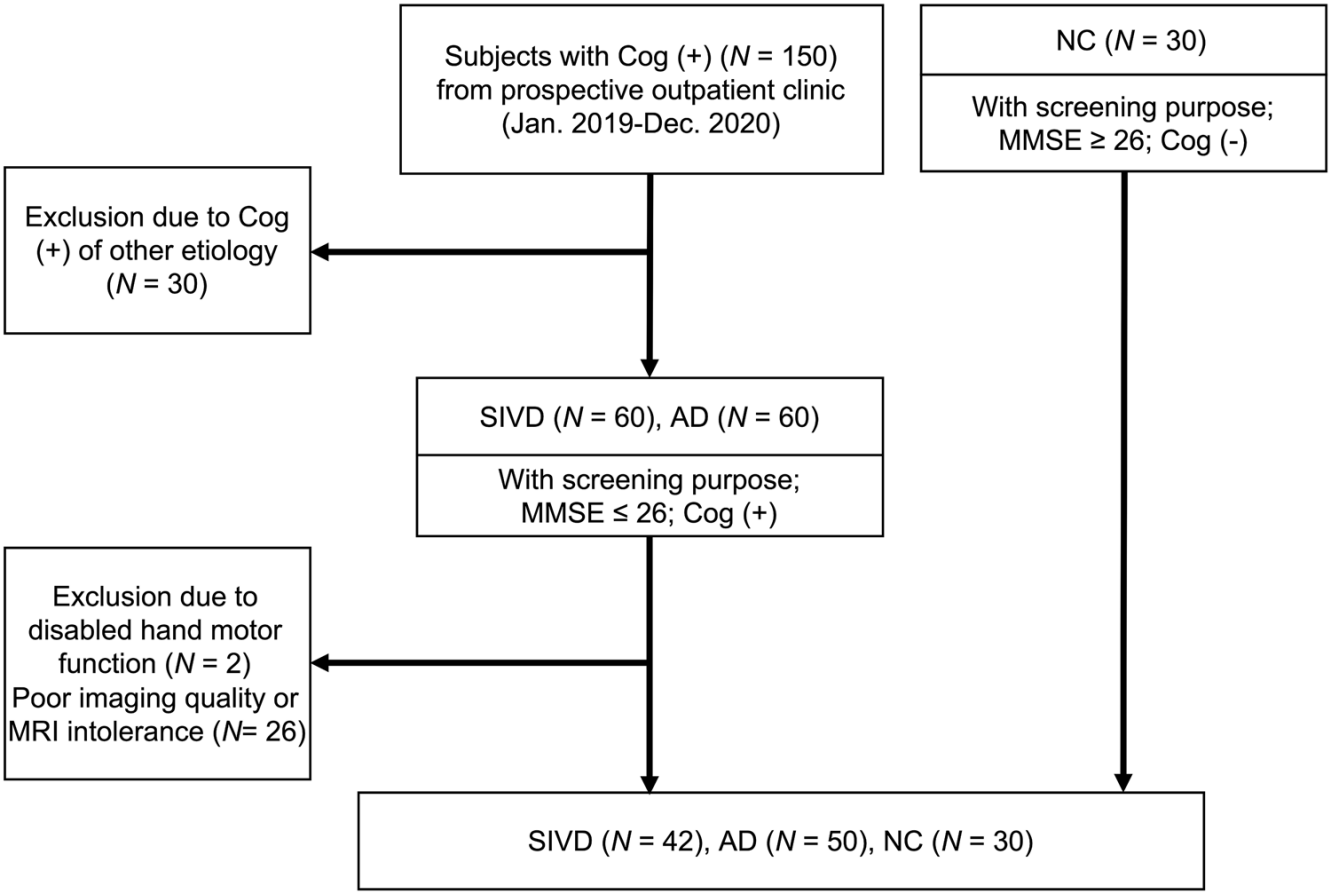
Flow Chart of Participants Selection. SIVD = Subcortical ischemic vascular disease; AD = Alzheimer's disease; NC = normal cognition. Cog = Cognitive symptom. MMSE = Mini-Mental State Examination; MRI = magnetic resonance imaging.

This prospective, observational, cross-sectional single-center study was approved by the Research Ethics Committee of Taichung Tzu Chi Hospital (#REC-107-28). Written informed consent was obtained from all participants. A total of 150 subjects with cognitive complaints from an outpatient dementia clinic were consecutively screened from January 2019 to December 2020 (Fig. 3). Those with clinical assessments showing cognitive complaints, brain CT showing no cortical encephalomalacia, and a MMSE score ≤ 26 were initially included; those with incomplete cognitive assessment and brain MRI scans were excluded. SIVD was diagnosed using the research criteria proposed by Erkinjuntti et al.^1^; AD was diagnosed using the National Institute of Neurological and Communicative Disorders and Stroke and the Alzheimer's Disease and Related Disorders Association criteria^20^; patients with mixed dementia were excluded if the Hachinski Ischemic Scale was 5–6^21^. As the published^8^, the inclusion and exclusion criteria were presented in Supplementary Table S4 Overall, 42 patients with SIVD and 50 with AD were recruited in this study. In addition, another 30 older adults who showed no cognitive symptoms and had an MMSE score ≥ 26 were recruited as the NC group. There is some overlap of these participants with a previously published article^22^, however the analysis approaches were different from the current study.

### Clinical data collection

We recorded age, gender, education, cognitive symptom duration, and Hachinski Ischemic Score. WMHs were rated according to Scheltens scale (a total of 12 subregions within the supra-tentorial parts)^23^ by a neurologist (M.C.T.) with 11 years of experience, and quantified using the Lesion Prediction Algorithm (LPA)^24^. The LPA results, morphology of the thalamus, and number of lacunes were visually assessed by the same neurologist. Global cognitive scores including the MMSE, CASI, and Clinical Dementia Rating were rated by a team of certified clinical psychologists led by Y.H.H., who has 8 years of experience.

### MRI protocols

The brain MR images were obtained at a 3 T MRI scanner (Discovery MR750, GE HealthCare, Milwaukee, WI) with an eight‐channel phased‐array head coil. The following protocols were acquired: three‐dimensional T1‐weighted imaging (3D‐T1), T2 fluid‐attenuated inversion recovery imaging (T2‐FLAIR), and diffusional kurtosis imaging (DKI). For 3D‐T1, a fast spoiled gradient echo with RF‐spoiling (FSPGR) was performed with repetition time (TR) of 7.90 ms, echo time (TE) of 3.06 ms, inversion time (TI) of 450 ms, flip angle of 12°, matrix size (MTX) of 240 × 240 × 160, achieving an isotropic voxel size of 1 mm^3^. Parameters for T2‐FLAIR were TR of 12,000 ms, TE of 120 ms, TI of 2200 ms, field‐of‐view (FOV) of 220 mm, MTX of 384 × 224, 21 slices with slice thickness (SL) of 5 mm. Spin‐echo diffusion-weighted echo‐planar imaging was used to obtain DKI datasets. A total of 30 diffusion gradient directions were obtained with two *b*‐values (1000 and 2000 s/mm^2^) along each direction, and 5 un-weighted images (b0, *b* = 0 s/mm^2^) were acquired, resulting a total of 65 volumes for DKI dataset. Other scanning parameters were TR of 6000 ms, TE of 68 ms, FOV of 240 mm, MTX of 96 × 96, 60 slices with SL of 2.5 mm, resulting an isotropic spatial resolution of 2.5 mm^3^.

### MRI analysis

#### Image processing

**Figure 4:**
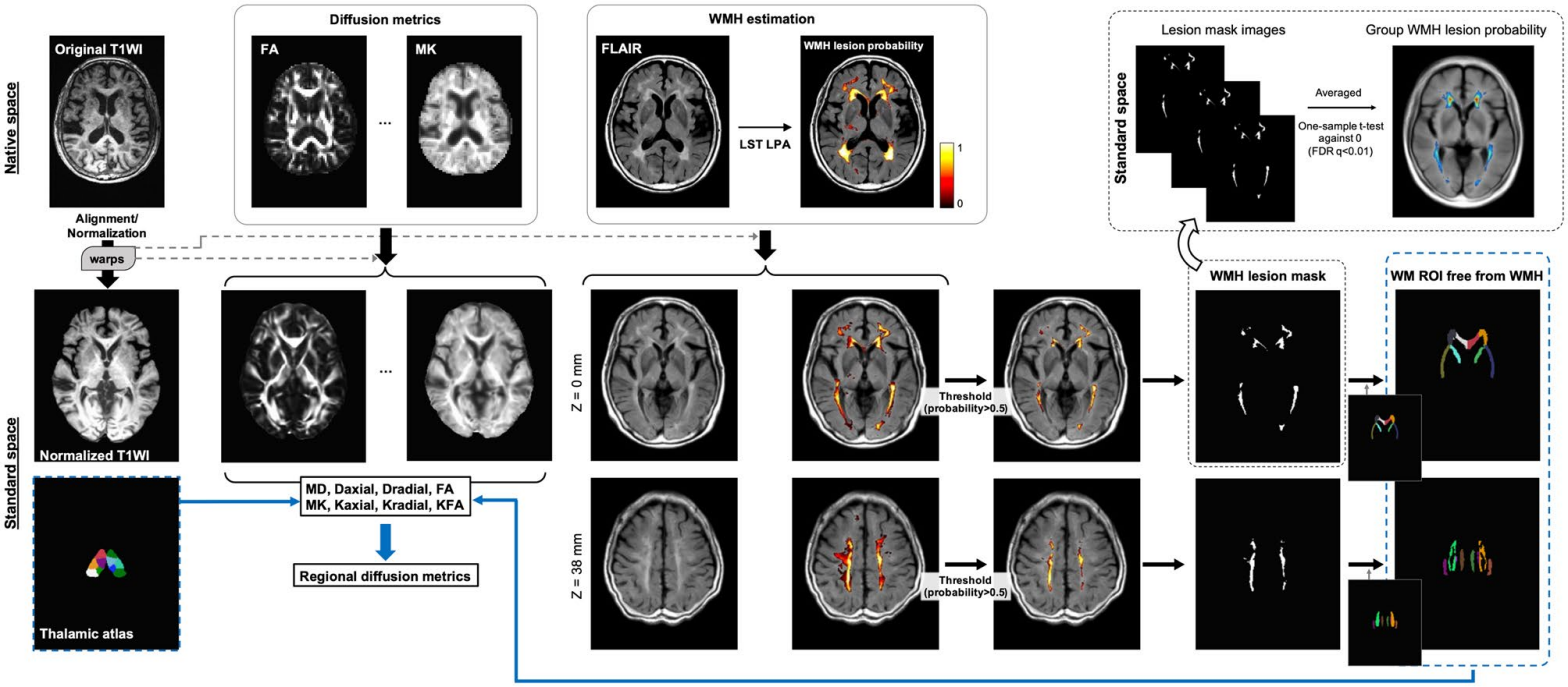
Illustration of the processing steps for MR images and the process for regional analysis. The diffusion metrics and the WMH probability maps were first estimated in native space, and consequently aligned on to standard MNI space according to the warp matrices from the co-registration process for T1WI (see Supplementary Materials for alignment details). The WMH maps were used to generated WMH lesion masks to calculate the WM ROIs free from WMH, and the WM ROIs free from WMH as well as the thalamic atlas were then utilized for the calculation of regional diffusion metrics. Group WMH lesion probability maps were also calculated as depicted in Supplementary Materials. T1WI = T1-weighted image. LST = Lesion Segmentation Tool. LPA = Lesion prediction algorithm. FDR = false discovery rate. WMHs = white matter hyperintensities. WM = white matter. FA = fractional anisotropy. MD = mean diffusivity. MK = mean kurtosis. KFA = kurtosis fractional anisotropy. Daxial = axial diffusivity. Dradial = radial diffusivity. Kaxial = axial kurtosis. Kradial = radial kurtosis.

Figure 4 illustrates the processing steps for MRI data (S.M.H. and L.W.K., with 12 years and 22 years of experience, respectively). All image processing and registration steps were performed using AFNI software (https://afni.nimh.nih.gov)^25^. All of the 3D-T1 images were aligned and normalized into standard MNI space, and all of the spatial warping transformation matrices were utilized for the alignment of the DKI-derived metrics^26,27^. Initially, the 3D-T1 image were skull-stripped and roughly registered with the MNI T1 template via 12-parameter affine alignment. After initial alignment, tissue segmentation was performed on the roughly-aligned T1 image to identify white matter, gray matter, and cerebrospinal fluid (CSF). CSF voxels were set to zero to generate the CSF-free T1 image. The same masking process was performed on the MNI T1 template to generate the CSF-free MNI T1 template. After this process, a two-step non-linear warping process was employed for better co-registration outcome. First, the CSF-free 3D-T1 image was co-registered to the CSF-free MNI T1 template by using non-linear co-registration, and this output non-linear warping transformation was then applied on the roughly-aligned 3D-T1 images to generate the first warped 3D-T1 image. The first warped 3D-T1 image was then non-linearly co-registered to the MNI T1 template again to form the final aligned 3D-T1 image. As the size of ventricle varies across subjects, the two-step procedure was carried out to achieve adequate image alignment results. All of the warp parameters and transformation matrices derived from the alignment process were used for the following alignment of diffusion weighted images (DWIs).

#### DKI reconstruction

The DKI data analyses were implemented using in-house MATLAB scripts (MathWorks, MA, USA). Before DKI reconstruction process, all the DWIs were denoised with local PCA method^28^. DKI reconstruction was performed according to the estimation approach of DKI model proposed by Tabesh et al*.*^29^. The DKI data was fit to the DKI model using the Levenberg–Marquardt algorithm with least-squared error estimations. The diffusivity and kurtosis metrics along all diffusion gradient directions were derived and averaged by using diffusion data with all b-values (i.e., 1000 and 2000 s/mm^2^). The quantitative metrics of DTI (MD; D_axial_; D_radial_; FA) and DKI (MK; K_axial_; K_radial_; KFA) were then calculated from the DKI model. The diffusion metrics were first estimated in each subject’s native space, and then the DKI parametric maps were aligned to MNI space according to the transformations from the following alignment process on DWIs. First, the b0 images were aligned to each subject’s own 3D-T1 images via 12-parameter affine alignment. Second, the T1-related transformation matrix and warp parameters described in previous section (‘Image processing’) were applied onto the T1-aligned b0 image to generate aligned b0 image in the MNI space. Finally, the DKI parametric maps were aligned to MNI space by applying the same transformations previously determined on b0 images. To reduce potential registration bias surrounding the edge of ventricular space and thalamus, a ventricle mask was generated from averaged b0 images of the SIVD group by AFNI software to avoid including unwanted ventricular voxels when calculating the regional DKI metrics.

#### WMH volume assessment

We performed automatic WMH segmentation by using the LPA^24,30^ as implemented in the lesion segmentation tool (LST 3.0.0, https://www.applied-statistics.de/lst.html) for SPM. This algorithm utilizes FLAIR images to estimate the lesion probability of white matter. The segmentation result of each subject was visually inspected by an experienced neurologist (M.C.T.). To calculate the total volume of WMH, we used LST’s default settings of probability threshold of 0.5 (probability of a voxel being WMH) (Supplementary Figure S1).

#### Regional diffusion metric

In the WMA, a total of 16 tracts including the corpus callosum (genu and body parts), the anterior limb of the internal capsule, anterior corona radiata, superior corona radiata, external capsule, cingulum (cingulate gyrus part), and superior longitudinal fasciculus of bi-hemispheres were used based on the JHU DTI-based white-matter atlases available in FSL (https://fsl.fmrib.ox.ac.uk/fsl/fslwiki/Atlases). In the WMA, pixels with WMHs are excluded according to the LPA method described above, and hence the measurements focus on WM *free* from WMHs. In the THA, a total of 14 thalamic sub-regions including the pulvinar, anterior, mediodorsal, ventral–lateral–dorsal, central, ventral-anterior, and ventral–lateral–ventral nuclei were used based on a segregated thalamic atlas^31^.

### Statistical analysis

Analysis of variance and the chi‐square test were used to compare demographic data. Analysis of covariance with Tukey post hoc comparison was used to compare diffusion metrics by controlling for age, education, symptom duration, volume of WMHs, and number of lacunes. After exploring the performance of diffusion metrics using discriminant analysis with clinical and macroscopic imaging constraints, receiver operating characteristic (ROC) curves were further used to examine the ability of the selected diffusion metrics to detect SIVD. The key goal of serial discriminant analyses aimed at identifying which diffusional kurtosis metric(s) perform the best value of correct classification. The purpose of additional analyses for examining both hemispheres or single hemisphere was to present the effect related to number of variables fed into the discriminant analyses as well as intra-group variation, if any. The rationale to include clinical and macroscopic imaging constraints was to examine the current diffusional kurtosis metrics among those population with a relatively early dementia stage (i.e., CDR ≤ 0.5) or limited cerebral vascular burden (i.e., WMH ≤ 15 ml and lacune number ≤ 8). To constrain composite diffusion metrics from multiple regions of interest, binary logistic regression was used to derive probability values of diffusion metrics in each ROC trial. Pearson correlation analysis was used to quantify relationships between diffusion metrics and cognitive parameters. Stepwise linear regression analysis was used to identify estimated effects of diffusion metrics on cognition. All statistical tests were performed using SPSS version 25 (IBM, Armonk, NY). A *P* value < 0.05 was considered statistically significant.

### Ethics declarations

This study was performed in line with the principles of the Declaration of Helsinki. Approval was granted by the Research Ethics Committee of our Hospital (#REC-107-28).

### Consent to participate

Informed consent was obtained from all individual participants included in the study.

### Supplementary Information

Supplementary Information.

## Data Availability

Data generated or analyzed during the study are available from the corresponding author on reasonable request.
