# Supplementary material for: Joint diffusional kurtosis magnetic resonance imaging analysis of white matter and the thalamus to identify subcortical ischemic vascular disease
Source: Sci Rep. 2024 Jan 31;14:2570. doi: 10.1038/s41598-024-52910-x (PMC10830492; doi:10.1038/s41598-024-52910-x)
Supplement: Supplementary file 1 — Supplementary Information. [file 41598_2024_52910_MOESM1_ESM.docx]

**Supplementary Materials**

**Joint diffusional kurtosis MRI analysis of white matter and the thalamus to identify subcortical ischemic vascular disease**

Min-Chien Tu, Sheng-Min Huang, Yen-Hsuan Hsu, Jir-Jei Yang, Chien-Yuan Lin, Li-Wei Kuo

Supplementary Figures

**Figure S1**. The averaged white matter hyperintensity (WMH) lesion probability maps of the three groups.

SIVD = Subcortical ischemic vascular disease; AD = Alzheimer's disease; NC = normal cognition. WMH=white matter hyperintensity.

Supplementary Tables

**Table S1.** Comparisons between metrics free from versus including white matter hyperintensities.

| **Metrics** | **White Matter Tracts** | |
| --- | --- | --- |
|  | Free from WMHs | Including WMHs |
| **Fractional Anisotropy** | 72.1 | 76.2 |
| **Kurtosis Fractional Anisotropy** | 66.4 | 68.0 |
| **Mean Diffusivity** | 76.2 | 80.3 |
| **Radial Diffusivity** | 71.3 | 77.0 |
| **Axial Diffusivity** | 76.2 | 77.9 |
| **Mean Kurtosis** | 77.9 | 80.3 |
| **Radial Kurtosis** | 74.6 | 77.0 |
| **Axial Kurtosis** | 68.9 | 65.6 |

Using discriminant analysis, correct classification rates of two methods are reported. The selected tracts including the corpus callosum (genu and body parts), and the anterior limb of internal capsule, anterior corona radiata, superior corona radiata, external capsule, cingulum (cingulate gyrus part), and superior longitudinal fasciculus of bi-hemispheres are used. WMH= white matter hyperintensities.

**Table S2.** Summary of the tracts and nuclei with the best statistical power in the optimized metrics (MK+ K_radial_+ FA).

|  | **SIVD vs. AD** | | **SIVD vs. NC** | | **AD vs. NC** | |
| --- | --- | --- | --- | --- | --- | --- |
|  | Region | AUC | Region | AUC | Region | AUC |
| **WMA** | Superior corona radiata_L | .880 | Anterior corona radiata_R | .934 | Cingulum (cingulate gyrus part)_R | .797 |
| **THA** | Ventral–lateral–ventral nuclei_L | .824 | Ventral–lateral–ventral nuclei _L | .912 | Central nuclei_L | .773 |

Regarding individual region of interest, the tracts and nuclei showing the best statistical power belongs to MK metrics. Anatomical region and their corresponding area under the receiver operating characteristic curve (AUC) are listed as above. R/L = Right/Left hemisphere. SIVD = Subcortical ischemic vascular disease; AD = Alzheimer's disease; NC = normal cognition. WMA= White matter atlas. THA= Segregated thalamus analysis. FA= fractional anisotropy. MK= mean kurtosis. K_radial_= radial kurtosis

**Table S3.** Estimated effect (ẞ coefficients) of diffusional kurtosis and diffusion tensor metrics on the Hachinski Ischemic Scale (*N* = 122).

|  | **White Matter Atlas** | | | | **Segregated Thalamus Analysis** | | | |
| --- | --- | --- | --- | --- | --- | --- | --- | --- |
| **Metrics** | **Regions** | **ẞ** | ***P*** | **95% CI** | **Regions** | **ẞ** | ***P*** | **95% CI** |
| **MK** | AIC_R | -.323 | <.001 | (-19.021,-5.831) | VLV_L | -.257 | .001 | (-11.679, -3.323) |
| **MD** |  |  |  |  | VLD_R | -.290 | .025 | (-6.875,-.468) |
|  |  |  |  |  | VLV_R | .188 | .049 | (.034,20.049) |
| **D_axial_** | AIC_L | .244 | .005 | (2.460,13.140) |  |  |  |  |
| **D_radial_** |  |  |  |  | VA_R | .434 | .001 | (2.663,10.725) |
| **FA** | AIC_R | -.243 | .001 | (-32.920,-8.125) |  |  |  |  |

Note.－Significant regions are reported by stepwise linear regression analysis, with independent variables including targeted imaging metrics showing significant correlation with the Hachinski Ischemic Scale. Metrics with a Variance inflation factor (VIF) ≥ 5 are removed. Estimated effect of all metrics is reported after controlling for age, education, symptom duration, the volume of white matter hyperintensities, and the number of lacunes. AIC= anterior limbs of the internal capsule. VLV= ventral latero-ventral. VLD = ventral latero-dorsal. VA= ventral-anterior. R/L = Right/Left hemisphere. FA= fractional anisotropy. MD= mean diffusivity. MK= mean kurtosis. D_axial_= axial diffusivity. D_radial_= radial diffusivity. Mean/axial/radial diffusivity variables are entered in units of 10^−3^mm^2^s^−1^.

**Table S4.** The inclusion and exclusion criteria for SIVD and AD in the current research.

| The Inclusion Criteria for SIVD* |
| --- |
| (1) cognitive complaints with interference in complex occupational and social activities  (2) evidence of subcortical ischemic changes in brain MRI  (3) Clinical Dementia Rating (CDR) = 0.5 ~ 1  (4) Mini-Mental State Examination (MMSE) score ≤ 26  (5) Hachinski Ischemic Scale score (HIS) ≥ 7 |
| The Inclusion Criteria for AD† |
| (1) changes in cognition reported by the patient, informant or clinician  (2) absence of profound subcortical ischemic change in brain MRI  (3) CDR = 0.5 ~ 1  (4) MMSE score ≤ 26  (5) HIS ≤ 4 |
| The Exclusion Criteria for SIVD and AD |
| (1) state of delirium  (2) stroke event within 2 weeks  (3) appearance of cortical and/or cortico-subcortical non-lacunar territorial infarcts and  watershed infarcts, hemorrhages, signs of normal pressure hydrocephalus, and specific  causes of white matter lesions (e.g. multiple sclerosis, sarcoidosis, brain irradiation)  (4) derangements in serology tests contributing to cognitive impairment (e.g. abnormal  levels of free T4, cortisol, folic acid, vitamin B12, or rapid plasma reagin)  (5) severe hearing or visual impairment |

The recruitment protocol follows the diagnostic criteria:

1. Erkinjuntti, T. et al. Research criteria for subcortical vascular dementia in clinical trials. Advances in dementia research, 23-30 (2000) (for SIVD)* [1]and
2. McKhann, G. M. et al. The diagnosis of dementia due to Alzheimer’s disease: Recommendations from the National Institute on Aging-Alzheimer’s Association workgroups on diagnostic guidelines for Alzheimer's disease. Alzheimer's & dementia 7, 263-269 (2011) (for AD)[2]†.

Supplementary references

1. Erkinjuntti, T., et al., *Research criteria for subcortical vascular dementia in clinical trials.* Advances in dementia research, 2000: p. 23-30.

2. McKhann, G.M., et al., *The diagnosis of dementia due to Alzheimer's disease: recommendations from the National Institute on Aging‐Alzheimer's Association workgroups on diagnostic guidelines for Alzheimer's disease.* Alzheimer's & dementia, 2011. **7**(3): p. 263-269.
